# Colonization of Germ-Free Piglets with Mucinolytic and Non-Mucinolytic *Bifidobacterium boum* Strains Isolated from the Intestine of Wild Boar and Their Interference with *Salmonella* Typhimurium

**DOI:** 10.3390/microorganisms8122002

**Published:** 2020-12-15

**Authors:** Alla Splichalova, Radko Pechar, Jiri Killer, Zdislava Splichalova, Vera Neuzil Bunesova, Eva Vlkova, Hana Subrtova Salmonova, Igor Splichal

**Affiliations:** 1 Laboratory of Gnotobiology, Institute of Microbiology, Czech Academy of Sciences, 549 22 Novy Hradek, Czech Republic; splichalova@gnotobio.cz (A.S.); zdispl@gnotobio.cz (Z.S.); 2 Department of Microbiology, Nutrition and Dietetics, Faculty of Agrobiology, Food and Natural Resources, Czech University of Life Sciences Prague, 165 00 Prague, Czech Republic; radko.pechar@seznam.cz (R.P.); killer@iapg.cas.cz (J.K.); bunesova@af.czu.cz (V.N.B.); vlkova@af.czu.cz (E.V.); salmonova@af.czu.cz (H.S.S.); 3 Department of Research, Food Research Institute Prague, 102 00 Prague, Czech Republic; 4 Institute of Animal Physiology and Genetics, Czech Academy of Sciences, 142 20 Prague, Czech Republic

**Keywords:** *Bifidobacterium boum*, *Salmonella* Typhimurium, goblet cells, mucin, mucinolytic, germ-free, gnotobiotic, piglet

## Abstract

Non-typhoidal *Salmonella* serovars are worldwide spread foodborne pathogens that cause diarrhea in humans and animals. Colonization of gnotobiotic piglet intestine with porcine indigenous mucinolytic *Bifidobacterium boum* RP36 strain and non-mucinolytic strain RP37 and their interference with *Salmonella* Typhimurium infection were compared. Bacterial interferences and impact on the host were evaluated by clinical signs of salmonellosis, bacterial translocation, goblet cell count, mRNA expression of mucin 2, villin, claudin-1, claudin-2, and occludin in the ileum and colon, and plasmatic levels of inflammatory cytokines IL-8, TNF-α, and IL-10. Both bifidobacterial strains colonized the intestine comparably. Neither RP36 nor RP37 *B. boum* strains effectively suppressed signs of salmonellosis. Both *B. boum* strains suppressed the growth of *S*. Typhimurium in the ileum and colon. The mucinolytic RP36 strain increased the translocation of *S*. Typhimurium into the blood, liver, and spleen.

## 1. Introduction

The coevolution of the host and its microbiota developed a mutually-beneficial relationship between them, and the microbial community became indispensable for the host’s metabolism and health [1]. The complex body microbiota consists of viruses [2], bacteria [3], archeae [4], fungi and yeasts [5], and protozoa [6]. Relationships between hosts and their microbiota can range from both-side convenient mutualism to host-destructive parasitism [7].

Balanced microbiota is an essential prerequisite for the host’s health, and its majority is located in the intestine [8,9]. The newborn intestine is colonized depending on the way of delivery, nutrition, and use of antibiotics [10,11]. Initially, the intestinal tract of vaginally delivered newborn colonize facultative anaerobes and create suitable conditions, e.g., lower pH and consumption of oxygen, for the subsequent onset of strict anaerobic species, e.g., *Bacteroides*, *Bifidobacterium*, and *Clostridium* spp. [10]. Breastfeeding refines the microbiota by additional microbes, human milk oligosaccharides (HMO), and immunological proteins [12], and the microbiome of breastfed infants consists of higher Proteobacteria and lower Bacteroidetes and Firmicutes than those formula-fed [13]. Bifidobacteria are the most abundant enteric bacteria in the vaginally born infant intestine [14,15]. They confer their beneficial effect by the saccharolytic activity toward glycans abundant in the infant intestine [16]. Moreover, they suppress pathogenic microorganisms [14,17,18,19]. In contrast, infants born through Cesarean section are mainly colonized with skin and hospital environment microbes, show lower numbers of bifidobacteria, and are more often colonized with *Clostridium difficile* [20]. Exclusively formula-fed infants are frequently colonized with *Escherichia coli*, *C. difficile*, *Bacteroides*, and *Lactobacillus* spp., compared with breastfed infants [21]. Early life represents an opportunity to shape the intestinal microbial ecosystem [12]. Adult-like microbiota forms in children at the age of 3–5 years and is individually characteristic and relatively stable for adult life [22]. The composition of the body microbiota varies in dependence on the host’s body surface and is highly personalized [23]. The most abundant bacteria are in the lower part of the intestinal tract that comprises in adults around 2000 species of predominantly anaerobic bacteria [24,25]. According to recently corrected counts, the total number of bacterial cells is comparable with the number of the host body cells [26].

A single layer of intestinal epithelial cells forms an interface between the intestinal lumen with bacteria and the host body. This interface must tolerate the luminal microbiota and their products and simultaneously protect the host against potentially harmful dietary antigens and invading pathogens [27]. The apical parts of the adjacent enterocytes of the epithelial layer are joined by tight junction proteins as claudins, occludin, and zonula occludens proteins and create a semipermeable layer [28]. This layer is covered by high molecular weight glycoprotein mucins that produce specialized enterocytes called goblet cells [27]. Mucins, as the main component of the mucus layer, protect the intestinal epithelium from infectious and non-infectious agents [29]. Mucin 2 (MUC2) is the predominant mucin in the human colon [30].

Much translational knowledge is obtained from experimental work with animal models that are mainly laboratory rodents in contrast that they are very dissimilar to humans in the transcriptome [31], microbiome composition [32,33], and duration of sepsis [34]. Humans and pigs share related anatomy, physiology, and genetics that predetermine the pig as a suitable animal model in human gastroenterology [35], infections [36], and sepsis [37]. Additionally, a similar composition of the porcine intestinal microbiome [33,38] incites the use of the pig in host-microbiota cross-talk translation research [35].

Gnotobiotic piglets proved their suitability for transplantation of the human microbiota [39,40]. Generally, the gnotobiotic animals are germ-free or animals associated with simple defined microbiota [41,42,43]. A crucial role for the host’s colonization resistance and its health plays the composition of the microbiota. Germ-free mice colonized with intestinal microbiota from the adult mice showed higher resistance to infection with *Salmonella* Typhimurium that their counterparts colonized with the intestinal microbiota from young mice [42]. A consortium of appropriately selected bacterial strains protected the mouse against infection with *S*. Typhimurium [41]. Knowledge of the interactions among the host, its microbiota, and pathogenic bacteria helps to establish strategies for modulation of the microbiota and its effective protection of the host against infectious diseases [44].

*Salmonella enterica* is one of the human and animal enteric pathogens. It consists of a group of over 2500 serovars [45]. A non-typhoidal *S*. Typhimurium belongs to the most worldwide spread serovars [46,47]. It causes enterocolitis (salmonellosis) in humans and pigs and affects mainly the distal ileum and colon [47,48]. Clinical signs of salmonellosis are fever, anorexia, diarrhea, vomiting, lethargy, and tremor. Salmonellosis is usually a self-limiting illness. However, it can endanger immunocompromised individuals by life-threatening systemic diseases [49].

Our work aimed to study a monocolonization of the intestine of newborn germ-free piglets with mucinolytic and non-mucinolytic strains of *Bifidobacterium boum*, its impact on the modification of the intestinal barrier, interference of bifidobacteria with *S*. Typhimurium, and its influence on bacterial translocation.

## 2. Materials and Methods

### 2.1. Ethics Statement

All animal experiments were reviewed and approved by the Animal Care and Use Committee of the Czech Academy of Sciences, protocol #117/2012.

### 2.2. Isolation of Wild Pig Indigenous Bifidobacteria

Bifidobacteria were isolated from the fresh fecal samples of 2 wild pigs using Wilkins–Chalgren agar (Oxoid, Basingstoke, UK) supplemented with soya peptone (5 g/L; Oxoid), mupirocin (100 mg/L), and glacial acetic acid (1 mL/L) in CO_2_/H_2_/N_2_ (10/10/80%) atmosphere in anaerobic jars with palladium catalysts (Oxoid). Cultures were grown in anaerobic Wilkins–Chalgren broth (Oxoid) supplemented with soya peptone (5 g/L; Oxoid) [50].

### 2.3. Identification and Characterization of Pig B. boum Strains

Bifidobacteria species were classified using 16S rRNA gene sequencing. For this purpose, the primers fD1 and rP2 [51] were employed. The EZBioCloud 16S rRNA gene-based ID database was used for classification based on the 16S rRNA gene sequence similarity [52]. Furthermore, particular strains were biochemically characterized by the API 50CHL test strips (BioMérieux, Marcy l’Etoile, France), allowing us to evaluate the ability to utilize 48 carbohydrate substrates [53].

### 2.4. Mucinolytic Activity of B. boum Strains

The mucinolytic activity of the *B. boum* strains was tested using a medium where porcine mucin (Sigma-Aldrich, St. Luis, MO, USA) was used as a selective factor [54]. Bifidobacterial cultures were grown in Wilkins–Chalgren broth (Oxoid) supplemented with soya peptone (5 g/L; Oxoid), were twice centrifuged (5000× *g* for 10 min), twice flushed with sterile saline, and resuspended in the PBS buffer. The cultures were inoculated (0.1%) into the broth with mucin and incubated at 37 °C for 48 h. Mucin utilization was accompanied by a change of bromocresol purple indicator (Sigma-Aldrich) from violet to yellow color as a consequence of reduced pH value through final products (mainly acetic and lactic acid) of specific fermentative metabolism of bifidobacteria. Cultivation was done under anaerobic conditions by displacing atmospheric oxygen with purified carbon dioxide.

### 2.5. Salmonella Typhimurium Strain LT2

*Salmonella enterica* serovar Typhimurium strain LT2 (*S*. Typhimurium or LT2) was from a collection of the microorganisms of the Institute of Microbiology of the Czech Academy of Sciences (Novy Hradek, Czech Republic). This *S*. Typhimurium strain was virulent for 1-week-old germ-free piglets [55].

### 2.6. Bacterial Suspensions

Fresh cultures of bacteria were prepared for each experiment by cultivation at 37 °C overnight. Bifidobacteria were cultivated anaerobically in vials containing 10 mL of Wilkins–Chalgren broth (Oxoid) supplemented with soya peptone (5 g/L, Oxoid). The cells were harvested by centrifugation at 4000× *g* for 10 min at RT, and the cell pellet was washed twice with 0.05 M phosphate buffer, pH 6.5 containing 500 mg/L cysteine (PBC). *S*. Typhimurium was cultivated on meat-peptone agar slopes (blood agar base; Oxoid). Bifidobacteria and *S*. Typhimurium were resuspended to 8 log CFU/mL density in PBC. The evaluated cell densities at 600 nm were verified by cultivation methods on appropriate agar. The cultivation of bifidobacteria was performed in 50 mm Petri dishes with Wilkins–Chalgren agar (Oxoid) supplemented with soya peptone (5 g/L, Oxoid), L-cysteine (0.5 g/L, Merck, Darmstadt, Germany), mupirocin (100 mg/L, Merck), and glacial acetic acid (1 mL/L, Merck). The Petri dishes were cultivated in an anaerostat at 37 °C for 48 h. *S*. Typhimurium was cultivated aerobically in a 90 mm Petri dish with MacConkey agar (Merck) at 37 °C for 24 h. The bifidobacteria and *S*. Typhimurium CFU were counted from the dishes optimally containing 10–100 colonies and 20–200 colonies, respectively.

### 2.7. Gnotobiotic Piglets

Germ-free piglets of miniature Minnesota based breed [56] (Animal Research Institute, Kostelec nad Orlici, Czech Republic) were obtained by hysterectomy on the 112th day of gestation. They were reared in fiberglass isolators with a heated floor, fed to satiety 6–7 times per day with cow’s milk-based formula by a nipple, and microbiologically tested as described in detail elsewhere [57].

### 2.8. Experimental Design

**Figure 1:**
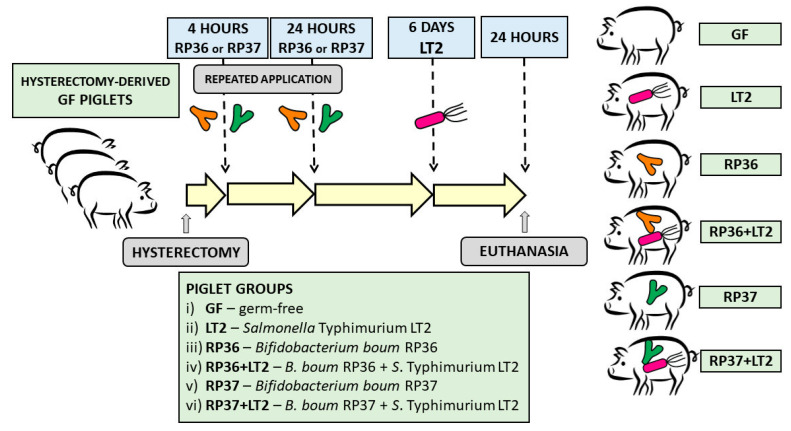
Schema of the experiment. The gnotobiotic piglets (*n* = 36) were assigned into six groups: (i) Germ-free (GF, *n* = 6); (ii) infected with *S.* Typhimurium strain LT2 for 24 h (LT2, *n* = 6); (iii) colonized with *Bifidobacterium boum* strain RP36 (RP36, *n* = 6); (iv) RP36 infected with *S*. Typhimurium for 24 h (RP36+LT2, *n* = 6); (v) colonized with *B. boum* strain RP37 (RP37, *n* = 6); (vi) RP37 infected with *S*. Typhimurium for 24 h (RP37+LT2, *n* = 6).

Each piglet group was composed of 3 independent hysterectomies. A total of 36 gnotobiotic piglets were assigned to 6 groups with 6 piglets per group (Figure 1): (i) Germ-free for the whole experimental period (GF); (ii) 1-week-old GF piglets orally infected with 6 log CFU of *S.* Typhimurium for 24 h (LT2); (iii) orally colonized with 8 log CFU of RP36 4 h and 24 h after hysterectomy (RP36); (iv) 1-week-old RP36 piglets orally infected with 6 log CFU of LT2 for 24 h (RP36+LT2); (v) orally colonized with 8 log CFU of RP37 4 h and 24 h after hysterectomy (RP37); (vi) 1-week-old RP37 piglets orally infected with 6 log CFU of LT2 for 24 h (RP37+LT2). The bacteria were applied orally in 5 mL of a milk diet, and GF piglets obtained 5 mL of milk only without bacteria. Twenty-four hours after the challenge with *Salmonella* (LT2, RP36+LT2, and RP37+LT2), the piglets were euthanized by exsanguination via cardiac puncture under isoflurane anesthesia. Their non-infected counterparts GF, RP36, and RP37, proceeded in the same way at the same age.

### 2.9. Clinical Signs

All piglets were examined for fever, anorexia, somnolence, and diarrhea during each feeding.

### 2.10. Bacterial Colonization of the GIT and Translocation

Samples of peripheral blood were cultivated log 10 diluted by PBC. Jejunum (40 cm of the proximal part of the jejunum) and ileum (40 cm segment of a distal part of the small intestine containing the distal jejunum and the ileum) were cut off, filled with 2 mL of PBC, gently kneaded, and rinsed. The whole colon was cut into small pieces on a 90 mm Petri dish and lavaged in 4 mL of PBC, 0.2 g of mesenteric lymph nodes, liver, and spleen were homogenized in 0.8 mL deionized water in a 2 mL Eppendorf tube containing 23.2 mm stainless steel beads in a TissueLyser LT beadbeater (Qiagen, Hilden, Germany). The lavages, tissue homogenates, and blood were serially diluted in PBC. The cultivation for bifidobacteria and *S*. Typhimurium was performed as the above-described for verification of CFU counts.

### 2.11. Blood Plasma

A citrated blood was centrifugated at 1200× *g* for 10 min at 8 °C. A protease inhibitor cocktail (Roche Diagnostics, Manheim, Germany) was added to plasma and the specimens were stored at −45 °C until followed processing.

### 2.12. Goblet Cells in the Ileum and Colon

The terminal ileum and colon specimens were fixed in Carnoy’s fluid for 30 min, dehydrated, and embedded in paraffin. Five µm cross-sections of tissue were stained with Alcian Blue and post-stained with Nuclear Fast Red (both Diapath, Martinengo, Italy) for mucin production. The preparates were examined under an Olympus BX 40 microscope with an Olympus Camedia C-2000 digital camera (Olympus, Tokyo, Japan). The number of mucin-producing goblet cells in the ileum and colon was counted as cells stained with Alcian Blue. The density of goblet cells per area of the tunica mucosa was counted.

### 2.13. Total RNA Isolation and Reverse Transcription

RNA was isolated and cDNA synthesized a described previously [43]. Briefly, the RNAlater stored cross-sections of the terminal ileum, and transversal colon was homogenized with 2 mm zirconia beads (BioSpec Products, Bartlesville, OK, USA) in TissueLyser LT beadbeater (Qiagen, Hilden, Germany) and total RNA isolated by the RNeasy Plus Mini kit (Qiagen). Of total RNA, 500 ng was reverse transcribed by QuantiTect Reverse Transcription kit (Qiagen). The synthesized cDNA was 1/10 diluted with PCR quality water (Life Technologies, Carlsbad, CA, USA), and these PCR templates were stored at −25 °C until the Real-Time PCR.

### 2.14. Real-Time PCR

Two µl of the PCR template was added to 18 µl of the FastStart Universal Probe Master (Roche Diagnostics) containing 500 nM each of the forward and reverse primers (Generi-Biotech, Hradec Kralove, Czech Republic) and 100 nM locked nucleic acid (LNA) probe (Universal ProbeLibrary; Roche Diagnostics) (Table 1). Amplification was performed in duplicates in 45 cycles with heating at 95 °C for 15 s and 60 °C for 60 s on an iQ cycler (Bio-Rad, Hercules, CA, USA). Relative mRNA expressions were calculated by the 2^-∆C^_T_ method [58] and normalized to β-actin and cyclophilin A by GenEx 6 software (MultiD Analyses AB, Gothenburg, Sweden).

### 2.15. Luminex xMAP Technology

Plasma levels of IL-8, TNF-α, and IL-10 were measured by a paramagnetic sphere-based xMAP technology (Luminex Corporation, Austin, TX, USA) with a Porcine ProcartaPlex kit (Affymetrix, Santa Clara, CA, USA) on the Bio-Plex and evaluated by Bio-Plex Manager 4.01 software (Bio-Rad, Hercules, CA, USA), as described previously [43].

### 2.16. Statistical Analysis

Differences among the groups in parameters with normal distribution were evaluated with one-way analysis of variance (ANOVA) with Tukey’s multiple comparisons post-hoc test. Values that did not meet the normal distribution were evaluated with Kruskal–Wallis with Dunn’s multiple comparisons post-hoc test. The statistical comparisons were performed at *p* ˂ 0.05 by GraphPad 6 software (GraphPad Software, San Diego, CA, USA) and statistical differences depicted in figures by a letter system.

## 3. Results

### 3.1. Isolation, Characterization, and Selection of Pig Indigenous Bifidobacterium boum Strains

In total, 17 strains of *B. boum* having the 16S rRNA gene similarities ≥99.71% to the *B. boum* type strain [59] were isolated from the fresh colonic samples of wild pigs. Then, all *B. boum* isolates were tested for mucinolytic activity, as described in previous studies [54,60]. *B. boum* designed RP36 was only one strain that showed mucinolytic activity, and its RP37 as its non-mucinolytic counterpart was taken for experiments. The RP36 strain revealed 99.71% and RP37 strain 99.85% 16S rRNA gene sequence similarity to the reference type strain. Their 16S rRNA gene sequences are deposited in the databases GenBank/EMBL/DDBJ under accession numbers MT742662 and MT742663, respectively. The resulted pattern was similar to the type strain of *B. boum* [61].

### 3.2. Clinical Signs of Salmonellosis

The germ-free (GF) and *B. boum* monocolonized non-infected piglets (RP36 and RP37) thrived. The piglets infected with *S*. Typhimurium LT2 (LT2, LT2+RP36, and LT2+RP37) suffered from anorexia, somnolence, fever, and non-bloody diarrhea.

### 3.3. Colonization of the Intestine with Bifidobacterium boum RP36 and RP37 Strains, Their Translocation to Mesenteric Lymph Nodes, Blood, Liver, Spleen, and Lungs and Interference with Salmonella Typhimurium

**Figure 2:**
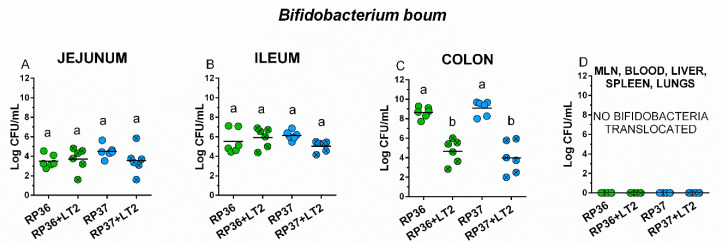
Colonization of the intestine of the gnotobiotic piglets with *B. boum* strains RP36 and RP37. *B. boum* strains RP36 and RP37 colony forming units (CFU) were counted in the jejunum (**A**), ileum (**B**), colon (**C**), mesenteric lymph nodes (MLN), blood, liver, spleen, and lungs (all (**D**)) in the *B. boum* monocolonized piglets (RP36 and RP37) and *B. boum* monocolonized piglets infected with *S*. Typhimurium (RP36+LT2 and RP37+LT2). Interferences between both *B. boum* strains (RP36 and RP37) and *S*. Typhimurium (RP36+LT2 and RP37+LT2) were evaluated by one-way ANOVA with Tuckey’s multiple comparisons post-hoc test. Statistical differences were marked by a letter system at *p* < 0.05. The same letter means no statistical significance. Log CFU are depicted as individual spots with mean as a horizontal line and *n* = 6 for all groups.

Both *B. boum* strains RP36 and RP37 were able to colonize the intestine of the gnotobiotic piglet in comparable densities around 4 log CFU/mL in the jejunum (Figure 2A), 6 log CFU/mL in the ileum (Figure 2B), and 9 log CFU in the colon (Figure 2C). No bacteremia or translocation into the mesenteric lymph nodes, liver, spleen, and lungs were observed (Figure 2D). The influence of *S*. Typhimurium on the growth of *B. boum* in the intestine was studied by comparison of the groups RP36 versus RP36+LT2 and RP37 versus RP37+LT2 (Figure 2A–C).

No differences between *B. boum* alone (RP36 and RP37) and in the presence of *S*. Typhimurium (RP36+LT2 and RP37+LT2) were found in the jejunum (Figure 2A) and ileum (Figure 2B). In the colon (Figure 2C), the presence of *S*. Typhimurium in RP36+LT2 and RP37+LT2 groups statistically significantly decreased the counts of both *B. boum* strains (Figure 2C). The presence of *S*. Typhimurium did not cause *B. boum* bacteremia or its translocation into observed organs (Figure 2D).

### 3.4. Colonization of the Intestine with Salmonella Typhimurium, Its Translocation to Mesenteric Mesenteric Lymph Nodes, Blood, Liver, Spleen, and Lungs, and Interference with Bifidobacterium boum RP36 and RP37 Strains

**Figure 3:**
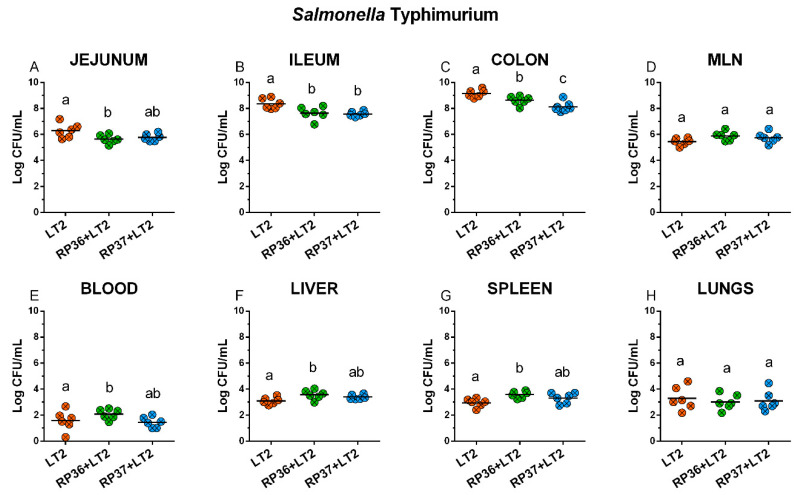
*S.* Typhimurium counts in the intestine, mesenteric lymph nodes (MLN), blood, liver, spleen, and lungs. *S*. Typhimurium strain LT2 (LT2) colony-forming units (CFU) were counted in the jejunum (**A**), ileum (**B**), colon (**C**), mesenteric lymph nodes (MLN; (**D**)), blood (**E**), liver (**F**), spleen (**G**), and lungs (**H**). *S*. Typhimurium (LT2) and its interferences with *B. boum* strains RP36 (RP36+LT2), and RP37 (RP37+LT2) were evaluated by one-way ANOVA with Tukey’s multiple comparisons *post-hoc* test. Statistical differences were marked by a letter system at *p* < 0.05. The same letter means no statistical significance. Log CFU are depicted as individual spots with mean as a horizontal line and *n* = 6 for all groups.

*Salmonella* Typhimurium counts reached around 6 log CFU/mL in the jejunum, 8 log CFU/mL in the ileum, 9 log CFU/mL in colon, 6 log CFU/g in mesenteric lymph nodes, 2 log CFU/mL in blood, and 3 log CFU/g in the liver, spleen, and lungs as it is depicted in Figure 3A–H. *Salmonella* growth was suppressed in the jejunum, but only in the case of RP36 was this suppression statistically significant. In the ileum (Figure 3B) and colon (Figure 3C), both *B. boum* strains suppressed *S*. Typhimurium counts statistically significantly. This suppression by RP37 strain was statistically significantly higher than by the RP36 strain (Figure 3C). Neither RP36 nor RP37 influences *Salmonella* translocation into the mesenteric lymph nodes (Figure 3D). Bacteremia of *S*. Typhimurium was statistically significantly higher in the presence of the RP36 strain but non-significantly lower than in the presence of the RP37 strain (Figure 3E). The differences between *Salmonella* counts in the presence of RP36 and RP37 *B. boum* strains were not significant. The same statistical relations, as in the case of bacteremia, were in translocations to the liver (Figure 3F) and spleen (Figure 3G). The *S*. Typhimurium counts in the lungs were not influenced by the presence of any *B. boum* strain (Figure 3H).

### 3.5. Density of Goblet Cells in the Ileum and Colon

**Figure 4:**
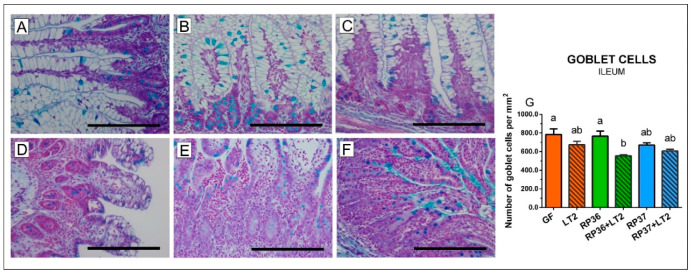
Goblet cells density in the ileum of gnotobiotic piglets: Germ-free (GF; (**A**)), colonized with *B. boum* RP36 (RP36; (**B**)), colonized with *B. boum* RP37 (RP37; (**C**)), infected with *S*. Typhimurium LT2 (LT2; (**D**)), colonized with RP36 and infected with LT2 (RP36+LT2; (**E**)), and colonized with RP37 and infected with LT2 (RP37+LT2; (**F**)). Bars represent 500 μm. The goblet cell counts (**G**) are presented as mean + SEM. Statistical differences were calculated by one-way ANOVA with Tukey’s multiple comparison post-hoc test, and *p*-values < 0.05 are denoted with different letters above the columns. Six samples in each group were analyzed.

The non-infected ileum of the GF, RP36, and RP37 groups were rich for vacuolized enterocytes (Figure 4A–C). The *Salmonella* infection shortened villi and reduced the number of the vacuolated enterocytes (Figure 4D–F). The infected ileum also showed edema and infiltration of neutrophils. The piglets colonized with mucinolytic *B. boum* RP36 and infected with *S*. Typhimurium (RP36+LT2) was the only group that showed a statistically significant decrease of the goblet cell density compare to the GF piglets (Figure 4G).

**Figure 5:**
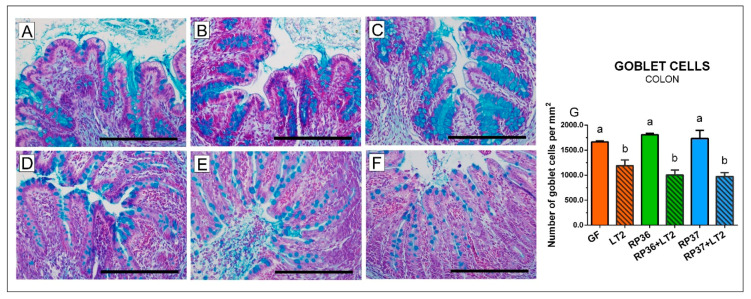
Goblet cells density in the colon of gnotobiotic piglets: Germ-free (GF; (**A**)), colonized with *B. boum* RP36 (RP36; (**B**)), colonized with *B. boum* RP37 (RP37; (**C**)), infected with *S*. Typhimurium LT2 (LT2; (**D**)), colonized with RP36 and infected with LT2 (RP36+LT2; (**E**)), and colonized with RP37 and infected with LT2 (RP37+LT2; (**F**)). Bars represent 500 µm. The goblet cell counts (**G**) are presented as mean + SEM. Statistical differences were calculated by one-way ANOVA with Tukey’s multiple comparison post-hoc test, and *p*-values < 0.05 are denoted with different letters above the columns. Six samples in each group were analyzed.

In the colon, bifidobacteria (Figure 5A–C) did not influence the number of mucin-producing cells in comparison with the GF group (Figure 5G). All *Salmonella*-infected groups (Figure 5D–F) showed statistically significantly lower goblet cell density (Figure 5G). Neither *B. boum* RP36 nor RP37 prevented a decrease of the goblet cell density (Figure 5E,F). Similar to the ileum, the infected colon was infiltrated with neutrophils.

### 3.6. Expression of Mucin 2 and Villin mRNA in the Ileum and Colon

**Figure 6:**
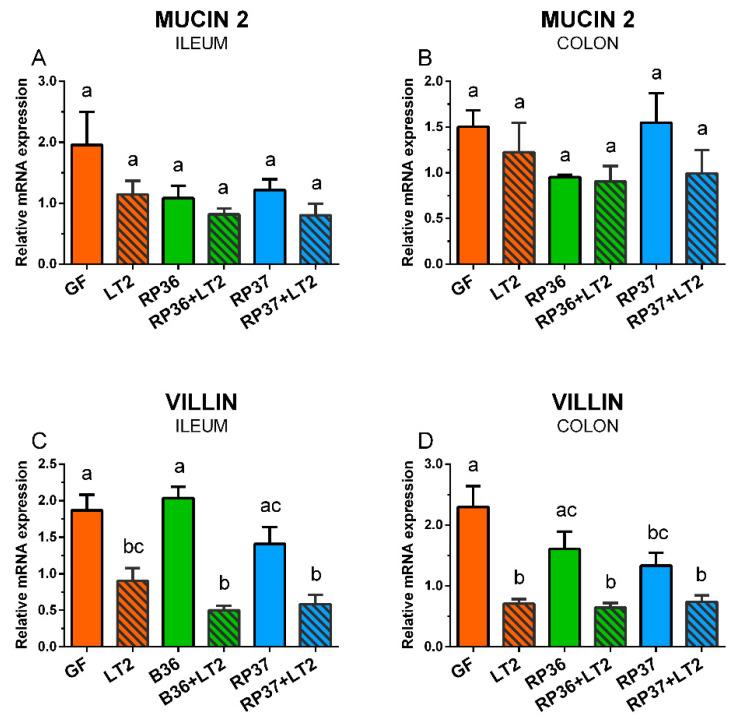
Expression of mucin 2 (**A**,**B**) and villin (**C**,**D**) mRNA in the ileum (**A**,**C**) and colon (**B**,**D**) of the gnotobiotic piglets: Germ-free (GF), infected with *S*. Typhimurium LT2 (LT2), colonized with *B. boum* RP36 (RP36), colonized with RP36 and infected with LT2 (RP36+LT2), colonized with *B. boum* RP37 (RP37), and colonized with RP37 and infected with LT2 (RP37+LT2). The values are presented as mean + SEM. Statistical differences were calculated by one-way ANOVA with Tukey’s multiple comparison post-hoc test, and *p*-values < 0.05 are denoted with different letters above the columns. Six samples in each group were analyzed.

The expression of mRNA for mucin 2 in the ileum and colon was influenced neither by colonization with *B. boum* strains nor infection with *S*. Typhimurium (Figure 6A,B). In contrast, the villin expression was statistically significantly reduced by infection with *S*. Typhimurium (Figure 6C,D). Neither *B. boum* strain RP36 nor RP37 prevented this reduction.

### 3.7. Expression of Tight Junction Protein mRNA in the Ileum and Colon

**Figure 7:**
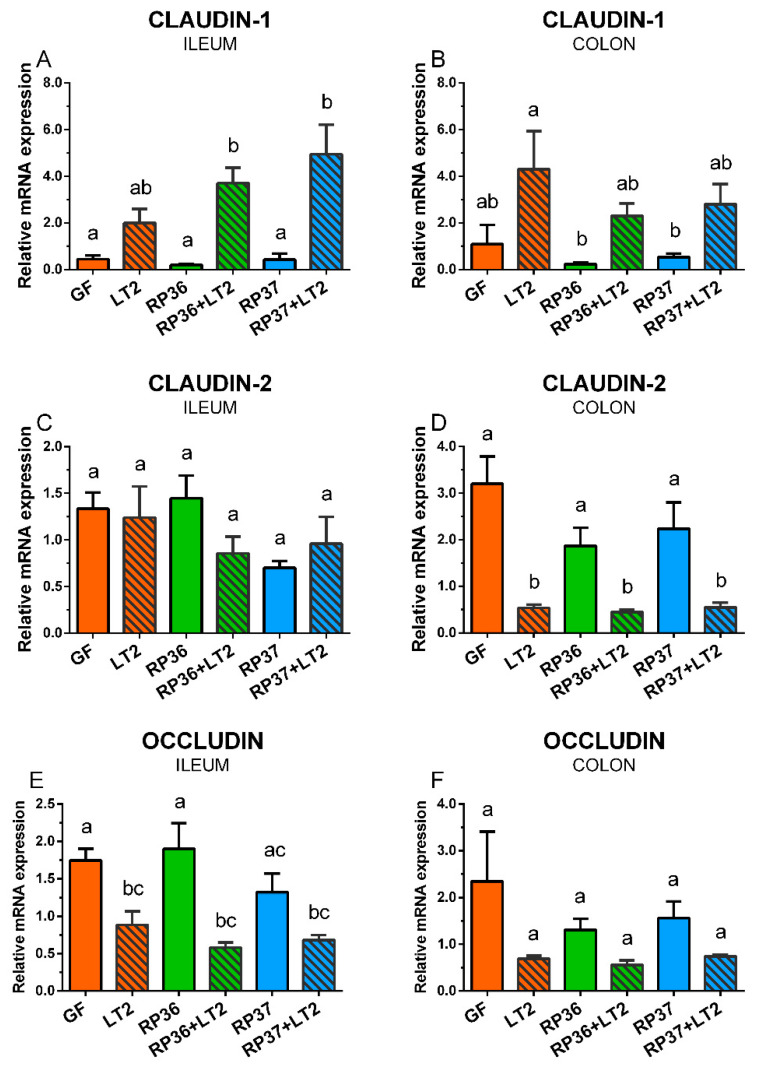
Expression of claudin-1 (**A**,**B**), claudin-2 (**C**,**D**), and occludin (**E**,**F**) mRNA in the ileum (**A**,**C**,**E**) and colon (**B**,**D**,**F**) of the gnotobiotic piglets: Germ-free (GF), infected with *S*. Typhimurium LT2 (LT2), colonized with *B. boum* RP36 (RP36), colonized with RP36 and infected with LT2 (RP36+LT2), colonized with *B. boum* RP37 (RP37), and colonized with RP37 and infected with LT2 (RP37+LT2). The values are presented as mean + SEM. Statistical differences were calculated by one-way ANOVA with Tukey’s multiple comparison post-hoc test, and *p*-values < 0.05 are denoted with different letters above the columns. Six samples in each group were analyzed.

Claudin-1 was upregulated by infection with *S*. Typhimurium in the ileum (Figure 7A). The increase induced by *S*. Typhimurium alone (LT2) was statistically non-significant, but both *B. boum* strains emphasized these changes in the RP36+LT2 and RP37+LT2 groups. A similar pattern of the *S*. Typhimurium stimulatory effect was observed in the colon, but without any statistical significance compared to the GF piglets (Figure 7B). The infection did not show any visible effect on the claudin-2 in the ileum (Figure 7C). However, the *Salmonella* infection downregulated the expression of claudin-2 in the colon of all *S*. Typhimurium-infected groups (Figure 7D). In contrast to claudin-1 in the ileum, occludin was statistically significantly downregulated by *S*. Typhimurium in comparison with the GF piglets (Figure 7E). No statistically significant differences in the occludin expression were found in the colon (Figure 7F).

### 3.8. Plasmatic Levels of Inflammatory Cytokines IL-8, TNF-α, and IL-10

**Figure 8:**
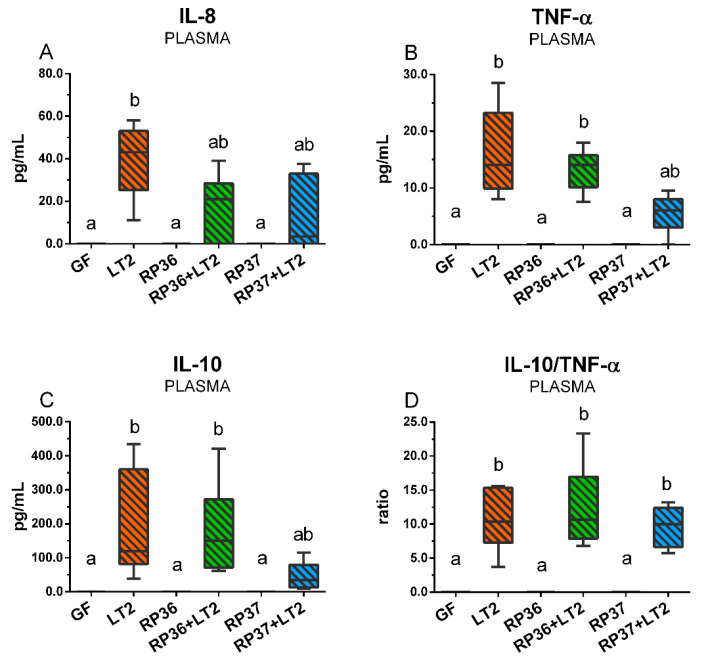
Plasmatic levels of IL-8 (**A**), TNF-α (**B**), IL-10 (**C**), and ratio IL-10/TNF-α (**D**) in the gnotobiotic piglets: Germ-free (GF), infected with *S*. Typhimurium LT2 (LT2), colonized with *B. boum* RP36 (RP36), colonized with RP36 and infected with LT2 (RP36+LT2), colonized with *B. boum* RP37 (RP37), and colonized with RP37 and infected with LT2 (RP37+LT2). The values are presented as boxes indicating the lower and upper quartiles, the central line is the median, and the ends of the whiskers depicts the minimal and maximal values. Statistical differences were calculated by the Kruskal-Wallis test with Dunn’s multiple comparison post-hoc test, and *p*-values < 0.05 are denoted with different letters above the columns. Six samples in each group were analyzed.

Plasmatic levels of IL-8 (Figure 8A) were not detectable in the GF, RP36, and RP37 groups. In contrast, they were induced in all groups infected with *S*. Typhimurium (LT2, RP36+LT2, and RP37+LT2). However, a statistically different increase was in the LT2 group only. Both piglet groups preliminary colonized with *B. boum* strains were not statistically different neither from the LT2 group nor in non-infected piglets of all three groups. TNF-α was significantly increased in the LT2 and RP36+LT2 groups but not in the RP37+LT2 group (Figure 8B). The differences among all LT2 infected groups were statistically non-significant. IL-10 showed the same statistical profile with differences among groups as TNF-α (Figure 8C). The ratio IL-10/TNF-α was statistically significantly increased in all *Salmonella*-infected groups (Figure 8D).

## 4. Discussion

Humans and pigs show high similarity in their gastrointestinal microbiota [33,38]. Therefore, the newborn piglets can be transplanted by human microbiota [40,62,63], and preterm piglets have been used as a model of vulnerable preterm infants [57,63,64]. Bifidobacteria are the most abundant members of newborn intestinal microbiota. They are obligatory anaerobes, and their growth in oxygen presence is limited [15]. Pigs are commonly inhabited by various bifidobacterial species [65,66]. It was proved that both orally simultaneously applied *Lactobacillus rhamnosus* GG and *Bifidobacterium animalis* subsp. *lactis* successfully colonized the sterile intestine of germ-free piglets [67]. Other experiments showed that indigenous porcine *Bifidobacterium choerinum*, even alone without the support of any other oxygen-consuming bacteria, colonized the intestine of the germ-free piglets [68]. We chose for our experiments *B. boum* for its typical presence in the intestinal tract of domesticated and wild pigs [59,61,65,66].

The bacteria-rich intestinal lumen and sterile host body are separated by a thin cell monolayer composed of enterocytes. This layer is covered by mucus that forms high molecular mass oligomeric glycoproteins referred to as mucins. Thus, the mucus is the first sentinel that protects the host against bacterial translocation. The mucus layer is continuously secreted and moves to clear trapped material and remove it from epithelial surfaces. The main secreted gel-forming mucin of the healthy human and mouse intestine is mucin 2 (MUC2) [30]. Mucinolytic ability rarely occurs among commonly beneficial bifidobacteria, with the exception of *Bifidobacterium bifidum* [60]. The absence of both mucinolytic activity and translocation were chosen as criteria confirming beneficial properties of probiotic *Bifidobacterium* *longum* BB536, *Bifidobacterium* *breve* M-16V, and *Bifidobacterium* *infantis* M-63 [69]. Therefore, we aimed to isolate typical porcine indigenous bifidobacterial strain but with mucinolytic activity. The isolated *B. boum* strain RP36 was only one of 17 tested *B. boum* strains that showed mucinolytic activity. The *B. boum* strain RP37 was taken into experiments as the RP36 non-mucinolytic counterpart. We were interested in how the modification of the mucus can influence the intestinal barrier and possible bacterial translocation as bifidobacteria, so *S*. Typhimurium.

Both *B. boum* strains were applied a short time after hysterectomy and on a subsequent day. They successfully and comparably colonized the jejunum, ileum, and colon of the colostrum-free germ-free piglets. However, neither *B. boum* strain RP36 nor strain RP37 translocated and caused bacteremia. It testified about the safety of both bifidobacterial strains [69] for the colostrum-free gnotobiotic piglets. In contrast to commonly beneficial bifidobacteria, *Salmonella enterica* serovar Typhimurium is an enteric pathogen, and its infection in humans and pigs is associated with enterocolitis [47,48]. *S*. Typhimurium is sophisticated bacteria that can manipulate the complex conventional microbiota composition to its profit [70]. The used strain LT2 of *S*. Typhimurium is often called “laboratory strain” [71]. It is almost avirulent for one-week-old conventional piglets [72] but lethal for germ-free piglets of the same age [55]. We found that bifidobacterial counts of both strains of *B. boum* in monocolonized piglets or in the piglets subsequently infected with *S*. Typhimurium were comparable in the jejunum and ileum, but they were diminished in the colon. *S*. Typhimurium stimulates the host to produce oxidizing metabolites that suppress the growth of strictly anaerobic bifidobacteria [15,73]. The colon is the main site of bifidobacteria protective action. This protective action consists mainly of acetic acid production that prevents infection with enteric pathogens [17]. Both mucinolytic RP36 and non-mucinolytic RP37 *B. boum* strains did not translocate to body organs in the presence of *S*. Typhimurium. Despite the fact that *Salmonella* counts were decreased in the intestine in the preliminary colonization of the germ-free piglets with both *B. boum* strains, all *Salmonella*-infected piglets suffered diarrhea. *S*. Typhimurium extensively translocated into the blood and spread to the liver and spleen. The preliminary colonization with the mucinolytic RP36 *B. boum* strain this translocation increased, but such effect was not observed in non-mucinolytic RP37 *B. boum* strain. The mucinolytic *B. boum* probably partially disrupted the intestinal barrier and facilitated *Salmonella* translocation [74,75].

We paid our attention to the mucin MUC2 that is the main secreted gel-forming mucin of the healthy human and mouse intestine. For this purpose, we used Alcian blue staining of acid mucins that are composed mainly of MUC2 [30]. The mucus is composed of two layers. The inner layer of the mouse colonic mucus is tightly attached to the epithelium and prevents bacterial translocation. In contrast, the outer layer is movable and allows colonization with bacteria but also their removal in the direction of intestinal peristalsis. Nevertheless, this mechanism can be disturbed by enteric infections [30,75]. In MUC2-/- mice, bacteria reached crypts that are normally covered by mucin and were in direct contact with the epithelial cells and induced inflammation and cancer in these mice. It points to the importance of a proprietary composition of MUC2 to participate in a mucus barrier and effectively separate bacteria from the epithelia [74]. In our experiments with gnotobiotic piglets, the infection with *S*. Typhimurium reduced the number of the goblet cells. This reduction was more obvious in the colon than the ileum. However, neither mucinolytic nor non-mucinolytic *B. boum* prevented this decrease. This reduction of goblet cells can probably be accounted by the expulsion of the cellular content of mucins as the response to infection [30,75]. To target more at MUC2, we analyzed changes in MUC2 mRNA. In contrast to the reduction of the acid mucin-producing goblet cells, we did not find any obvious influence of the *Salmonella* infection to MUC2 mRNA expression. Thus, we speculate that pre-prepared mucins are stored in goblet cells and continuously released. It takes a longer time to activate de novo synthesis than it was possibly found within 24 h of experimental infection because the goblet cells contain mucin for immediate usage if needed [30].

To analyze possible changes in the enterocyte layer and intestinal barrier, we analyzed mRNA expression of cytoskeletal protein villin and tight junction proteins claudin-1, claudin-2, and occludin. This barrier can be disrupted directly by enteric pathogens as *Salmonella* [76] or by their toxins [77]. Villin is associated with the microvillar actin filaments in the apical part of the intestinal epithelial cell brush border. It is a marker of enterocyte differentiation [78], participates in the restitution of damaged epithelia [79], and its expression increases as cells migrate from the crypt to the top of villi [78]. The apical side of the enterocyte extends to the tight junctions (TJ) created by TJ proteins, e.g., claudins and occludin. They form a band around the membrane creating a semipermeable joining between adjacent epithelial cells [28]. Villin and disrupted TJ facilitate the initial steps of *Salmonella* invasion [77,79], and *Salmonella* can translocate via lymph vessels or blood to the liver, spleen, and other organs [80]. The tight junctions *per se* enable paracellular absorption of nutrients and water and prevent the host against bacterial translocation as well as penetration of their toxins to the body [77]. The upregulated claudin-1 in both the ileum and colon of the gnotobiotic piglets can be an attempt to seal the intestinal barrier to prevent leakage of water and electrolytes from the body [81]. The opposite trend was found in the colon but not in the ileum for claudin-2. Its colonic transcription was downregulated in the piglets infected with *S*. Typhimurium. This difference can explain distinct managing with water and electrolytes in the ileum and colon [82] and different functions of claudin-1 and claudin-2 as representatives of the barrier-forming and pore-forming claudins, respectively [28]. In contrast to claudins, occludin participates in macromolecule flux [83] and cell migration [84]. The infection of mice with enterotoxigenic *Escherichia coli* downregulated occludin mRNA in the jejunum [85] as the infections with *S*. Typhimurium in our experiments. *S*. Typhimurium altered TJ by dephosphorylation of occludin by the induction of inflammatory cytokines tumor necrosis (TNF)-α. This alteration of the intestinal barrier resulted in 10 times higher *Salmonella* translocation and almost 2 times increased recruitment of neutrophils [86]. Similarly, *S*. Typhimurium induced neutrophil recruitment to the inflamed intestine in streptomycin-treated mice [87]. The neutrophil recruitment in the intestine of gnotobiotic piglets induced by avirulent rough mutants with incomplete lipopolysaccharide (LPS) of *S*. Infantis strain 1326/28 [88] or *S*. Typhimurium strain SF1591 [89] protected the piglets against subsequent infection with virulent *S*. Typhimurium F98 or *S*. Typhimurium strain LT2, respectively. The relation of virulence and LPS completeness in the gnotobiotic piglets was reported [55,90]. Lowered virulence of rough *Salmonella* mutants can reflect their lowered ability to bind to mucus and penetrate deeply, as was documented in experiments with streptomycin-treated mice [91].

Cytokines are important for the normal physiological processes of the host organism [92]. However, their exacerbated unregulated production upon some stimulus, e.g., infection or mechanical trauma, can inflict multiple organ dysfunction and host death [93,94]. The elevated systemic levels of inflammatory cytokines are used as markers of infection and sepsis [95]. Inflammatory cytokines, e.g., TNF-α change the permeability of the intestinal barrier by alteration of TJ protein expression [77]. We analyzed systemic levels of cytokines with different actions—chemotactic interleukin (IL)-8 that attracts neutrophils into the inflammatory site and activate them, TNF-α, which is a potent mediator of inflammation and immunoregulatory IL-10 that prevents an excessive inflammatory reaction [96]. The used cytokines are suitable to indicate the severity of enteric infections in the gnotobiotic pigs [97], and their plasmatic levels dramatically increased in the gnotobiotic piglets infected with necrotoxigenic *Escherichia coli* O55 [98] that obviously suffered from infection compare to their counterparts that thrived [99]. The overproduction of IL-10 was the main predictor of severity and fatal outcome of sepsis [96,100], and an increased ratio of IL-10/TNF-α was found in non-survived human patients [100]. The appearance of detectable levels of IL-10 in plasma also predicts a bad prognosis of surviving in the piglets [55,68,99]. The infection with *S*. Typhimurium induced the appearance of all inflammatory cytokines in blood plasma and allowed to count the IL-10/TNF-α ratio in contrast to all groups of non-infected piglets with nonmeasurable cytokines. The colonization with *B. boum* strains did not prevent the appearance of detectable levels of the analyzed inflammatory cytokines, but the colonization with *B. boum* RP37 prevented the increase plasmatic IL-10 to the comparable levels in both other *Salmonella*-infected piglet groups. This alleviating effect of *B. boum* RP37 may be important for the outcome of the infection with the *S*. Typhimurium LT2 strain.

## 5. Conclusions

Bifidobacteria are an important component of complex intestinal microbiota that can increase the resistance of the immunocompromised host to enteric infections. Therefore, we colonized the gnotobiotic piglets with intestinal barrier-disrupting mucinolytic *B. boum* strain RP36 and harmless non-mucinolytic strain RP37. Both *B. boum* strains reduced *S*. Typhimurium counts in the intestine, but the mucinolytic RP36 strain increased *S*. Typhimurium bacteremia and its translocation to the liver and spleen that was facilitated probably by disruption of the mucus layer, changes in the tight junctions, and the epithelial layer remodeling. Sepsis caused by *S*. Typhimurium was evaluated by the systemic levels of the analyzed inflammatory cytokines. Other studies are needed to induce a higher protective effect of the newborn intestine’s bacterial colonization with defined bacteria to increase resistance against infection with enteric pathogens.

## Figures and Tables

**Table 1:** LNA probe-based Real-Time PCR systems.

Gene	5′-forward primer-3′	5′-reverse primer-3′	#LNA Probe
BACT ^1^	TCCCTGGAGAAGAGCTACGA	AAGAGCGCCTCTGGACAC	9
CYPA ^2^	CCTGAAGCATACGGGTCCT	AAAGACCACATGTTTGCCATC	48
VILLIN	GCATGAAGAAGGTGGAGACC	ACGTTCCTCTTGCCCTTGA	42
CLD-1 ^3^	CACCACTTTGCAAGCAACC	TGGCCACAAAGATGGCTATT	3
CLD-2 ^4^	CTCGCGCCAAAGACAGAG	ATGAAGATTCCACGCAACG	77
MUC2 ^5^ OCLN ^6^	CCTGCTGCAAGGAGTATCG AAAGAGCTCTCTCGACTGGATAAA	TCTCGATGTGGGTGTAGGTG AGCAGCAGCCATGTACTCTTC	20 42

^1^ β-actin, ^2^ cyclophylin A, ^3^ claudin-1, ^4^ claudin-2, ^5^ muxin 2, ^6^ occludin.
